# Complexed trace mineral supplementation alters antioxidant activities and expression in response to trailer stress in yearling horses in training

**DOI:** 10.1038/s41598-021-86478-7

**Published:** 2021-04-01

**Authors:** Christine M. Latham, Emily C. Dickson, Randi N. Owen, Connie K. Larson, Sarah H. White-Springer

**Affiliations:** 1 Texas A&M Department of Animal Science, Texas A&M AgriLife Research, College Station, TX 77843 USA; 2 Zinpro Corporation, Eden Prairie, MN 55344 USA

**Keywords:** Mitochondria, Metabolism

## Abstract

To test the hypothesis that complexed trace mineral supplementation would increase antioxidant capacity and decrease muscle oxidative stress and damage in young horses entering an exercise training program, Quarter Horses (mean $\pm$ SD; 9.7 ± 0.7 mo) balanced by age, sex, and BW were assigned to receive complexed (**CTM**; n = 8) or inorganic (**INORG**; n = 8) trace minerals at -12 week relative to this study. Blood and muscle samples were collected before (week 0) and after 12 week of light exercise training surrounding a 1.5-h trailer stressor. Muscle glutathione peroxidase (GPx) activity was higher for CTM than INORG horses (*P* ≤ 0.0003) throughout the study. Following both trailer stressors, serum creatine kinase increased (*P* < 0.0001) and remained elevated through 24 h post-trailering (*P* < 0.0001). At week 0, muscle malondialdehyde, expression of *superoxide dismutase 2*, and whole blood GPx activity increased (*P* $\le$ 0.003) following trailering but trailering did not affect these measures at week 12. Young horses supplemented with CTM had higher muscle GPx activity than horses receiving INORG, but CTM did not affect damage markers following a stressor. Dietary CTM may be useful for improving antioxidant capacity during exercise training in young equine athletes.

## Introduction

Young equine athletes experience several stressors as they enter training and competition. Acute exercise bouts and trailer transportation between competitions or training facilities may cause an increase in mitochondrial production of reactive oxygen species (**ROS**), which can be deleterious to skeletal muscle health if not properly sequestered by antioxidant enzymes. Dietary complexed trace mineral supplementation has been shown to reduce oxidative stress in food animal models^1–3^. Dietary proteinate complexed-Zn supplementation increased glutathione peroxidase (**GPx**) and superoxide dismutase (**SOD**) activities in the spleen of barrows^4^. Additionally, biocomplexed minerals Fe, Mn, Zn, and Cu enhanced SOD and GPx activities in the liver of grower-finisher pigs^5^ and decreased plasma malondialdehyde (**MDA**), a measure of oxidative stress, in broilers^1^.

In horses, research on the effects of dietary organic trace mineral supplementation is limited. One study in horses demonstrated Cu-Lys may be better absorbed than CuSO_4_^6^. However, in the same group of horses, Cu-Lys and Zn-Met supplementation did not seem superior to CuSO_4_ and ZnSO_4_ in improving erythrocyte SOD activity during a 1-h standardized exercise test and immediately after recovery in horses^7^. It should be noted that exercise itself did not induce a change in SOD activity in that study, suggesting the exercise did not significantly increase systemic oxidative stress^7^. Additionally, supplemented mineral intakes of Cu-Lys and Zn-Met were higher than CuSO_4_ and ZnSO_4_, making interpretation of the results difficult.

In the present study, horses were maintained on a diet containing either complexed or inorganic trace minerals for 12 weeks, and then light exercise training was initiated. Horses were maintained on their respective diets and underwent the light exercise training program with blood and muscle samples from the triceps brachii (TB) and gluteus medius (GM) collected surrounding trailer stressors before (week 0) and after 12 week of exercise training. Our objective was to test the hypothesis that complexed trace mineral supplementation would increase systemic and muscle antioxidant gene expression and enzyme activities, and decrease oxidative stress and muscle damage in response to trailering stress in yearling Quarter Horses enrolled in a submaximal exercise training program.

## Results

### Responses to time, diet and exercise training

#### Growth

Body weight, heart girth, body length, wither height and hip height increased by week 12 (Supplemental Table S1*P* < 0.0001) as expected with normal growth. With exercise training, body condition score decreased from 6.1 at week 0 to 5.5 at week 12 (Supplemental Table S1; *P* < 0.0001). Body length (colt 149 ± 1 cm; filly 145 ± 1 cm) and body weight (colt 321 ± 6 kg; filly 303 ± 7 kg) tended to be greater for colts than fillies (*P* ≤ 0.07) but sex did not affect any other growth measurements (*P* > 0.10). No growth measurements were affected by diet or the diet $\times$ time interaction.

#### Oxidative stress and muscle damage

A diet $\times$ time interaction (*P* = 0.04) revealed that muscle MDA concentrations were greater for INORG than CTM horses at week 0 (*P* = 0.05). However, MDA decreased from week 0 to 12 in INORG horses (*P* = 0.006; Table 1) but remained unchanged in CTM horses, resulting in similar MDA levels between dietary treatment groups at week 12. Concentrations of MDA were greater in the TB than the GM for both dietary treatment groups throughout the study (*P* = 0.0002).

**Table 1:** Pre-trailer muscle malondialdehyde (MDA) concentrations in the gluteus medius (GM) and triceps brachii (TB) and serum creatine kinase (CK) activity at 0 and 12 weeks of submaximal exercise training in yearling horses supplemented with either complexed trace minerals (CTM; n = 8) or inorganic trace minerals (INORG; n = 8).

Variable	Muscle	Diet	Week		SEM	*P* value			
			0	12		Muscle	Diet	Time	Muscle $\times$ Diet $\times$ Time
*Muscle*									
MDA concentration (pmol/mg protein)	GM	CTM	56^#^	50^#^	7	0.0002	0.486	0.066	0.394
		INORG	58^#^	40*^#^					
	TB	CTM	60	69					
		INORG	85	65*					
*Serum*							Diet	Time	Diet $\times$ Time
CK activity (units/L)	–	CTM	83	73*	5		0.095	0.012	0.438
		INORG	77	59*					

*Within a row, mean differs from week 0 (*P* ≤ 0.05).

^#^Within a column, GM differs from TB (*P* ≤ 0.05).

Resting serum CK activity tended to be greater for CTM than INORG horses (*P* = 0.09) throughout the current study (Table 1). Pre-trailer serum CK activity decreased from week 0 to 12 (*P* = 0.01; Table 1) for both treatment groups.

#### Antioxidant activities

Overall, GPx activity was greater for CTM than INORG horses in both muscle groups (*P* = 0.002), and was greater in the TB than the GM (*P* = 0.007) for both treatments, but was not affected by time (Table 2). A main effect of time (*P* = 0.05) showed that muscle SOD activity increased from week 0 to 12. However, a trend for an effect of the diet $\times$ time interaction on resting muscle SOD activity (*P* = 0.08) revealed that SOD activity increased from week 0 to 12 in CTM (*P* = 0.007) but did not change in INORG horses. Neither resting whole blood GPx nor SOD activities were affected by time, diet, or their interaction (Table 2).

**Table 2:** Pre-trailer muscle glutathione peroxidase (GPx) and superoxide dismutase (SOD) activities in the gluteus medius (GM) and triceps brachii (TB), and whole blood GPx and SOD activities at 0 and 12 weeks of submaximal exercise training in yearling horses supplemented with either complexed trace minerals (CTM; n = 8) or inorganic trace minerals (INORG; n = 8).

Variable	Muscle	Diet	Week		SEM	*P* value			
			0	12		Muscle	Diet	Time	Muscle × Diet × time
*Muscle*									
GPx activity (nmol • min^−1^ • mg protein^−1^)	GM	CTM	12.72^a#^	11.06^a#^	2.04	0.007	0.002	0.415	0.963
		INORG	9.19^b#^	5.73^b#^					
	TB	CTM	17.65^a^	17.91^a^					
		INORG	8.89^b^	10.31^b^					
SOD activity (nmol • min^−1^ • mg protein^−1^)	GM	CTM	2.03^#^	3.00*^#^	0.44	0.001	0.627	0.043	0.559
		INORG	2.73^#^	2.50^#^					
	TB	CTM	2.81	3.78*					
		INORG	3.36	3.74					
*Whole blood*							Diet	Time	Diet $\times$ Time
GPx activity (nmol • min^−1^ • mg protein^−1^)	–	CTM	20.57	22.46	3.92		0.190	0.698	0.852
	–	INORG	24.77	25.43					
SOD activity (nmol • min^−1^ • mg protein^−1^)	–	CTM	0.21	0.23	0.03		0.284	0.804	0.507
	–	INORG	0.18	0. 18					

*Within a row, mean differs from week 0 (*P* ≤ 0.05).

^a,b^Within a column, differing letters indicate CTM differs from INORG within the specified variable (*P* ≤ 0.05).

^#^Within a column, GM differs from TB (*P* ≤ 0.05).

#### Muscle antioxidant gene expression

Pre-trailer *SOD1* expression was greater in the GM than the TB (*P* = 0.04) but was not affected by diet, time or any interactions (Table 3). A time $\times$ muscle group interaction (*P* = 0.0004) showed that pre-trailer *SOD2* expression was greater in the TB than the GM at week 0 (*P* < 0.0001) but increased in the GM by week 12 (*P* = 0.003; Table 3). A diet $\times$ muscle group interaction (*P* = 0.05) indicated that *SOD2* expression was greater for CTM than INORG horses in the GM (*P* = 0.02), and was greater in the TB than the GM for INORG horses (*P* = 0.0009) but was not different between muscle groups in CTM horses.

**Table 3:** Pre-trailer muscle *Cu–Zn superoxide dismutase* (*SOD1*) and *Mn superoxide dismutase* (*SOD2*) expression in the gluteus medius (GM) and triceps brachii (TB) at 0 and 12 weeks of submaximal exercise training in yearling horses supplemented with either complexed trace minerals (CTM; n = 8) or inorganic trace minerals (INORG; n = 8).

Variable	Muscle	Diet	Week		SEM	*P* value			
			0	12		Muscle	Diet	Time	Muscle $\times$ Diet $\times$ Time
*SOD1* (40-ΔCq)	GM^#^	CTM	39.90	39.86	0.10	0.013	0.252	0.545	0.063
		INORG	39.89	39.97					
	TB	CTM	39.67	39.67					
		INORG	39.82	39.77					
*SOD2* (40-ΔCq)	GM	CTM	39.46^a,y^	39.78^b,y^*	0.10	0.005	0.196	0.177	0.992
		INORG	39.26^a,x^	39.56^b,x^*					
	TB	CTM	39.74^b,y^	39.63^b,y^					
		INORG	39.82^b,y^	39.69^b,y^					

^#^Within a variable, GM differs from TB (*P* ≤ 0.05).

*Within a row, mean differs from week 0 (*P* ≤ 0.05).

^a,b^Means with different letters differ (*P* ≤ 0.05) due to muscle $\times$ time interaction (*P* = 0.0004).

^x,y^Means with different letters differ (*P* ≤ 0.05) due to muscle $\times$ diet interaction (*P* = 0.05).

### Responses to trailer stressors

#### Oxidative stress and muscle damage

**Figure 1:**
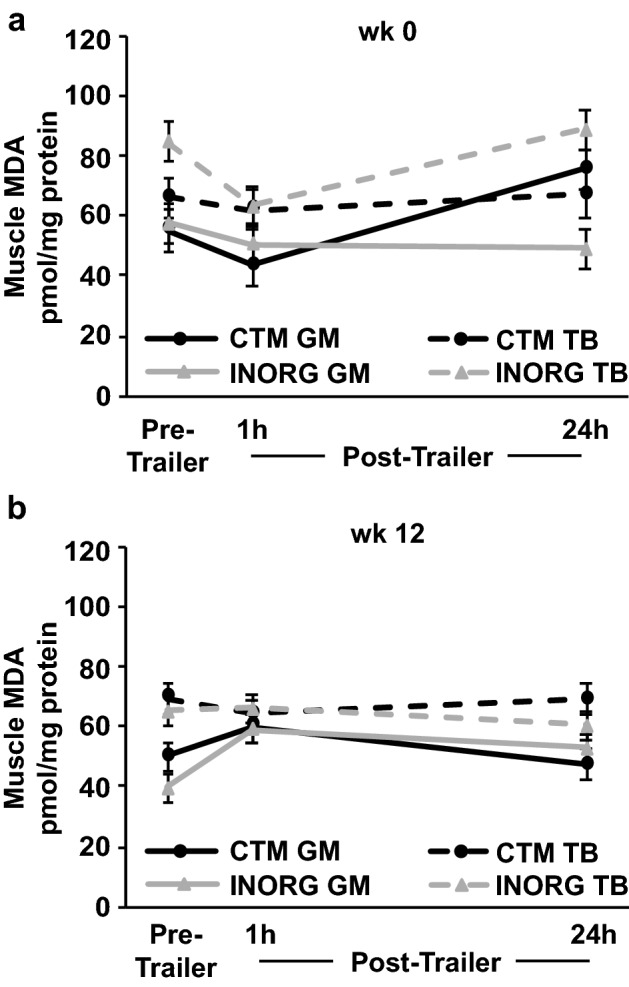
Muscle malondialdehyde (MDA) concentration before (Pre-Trailer), and 1 (1 h) and 24 h (24 h) after a 1.5-h trailer stressor. Trailer stressors occurred before (week 0; **a**) and after (week 12; **b**) 12 weeks of submaximal exercise training in yearling horses supplemented with either complexed trace minerals (CTM; n = 8) or inorganic trace minerals (INORG; n = 8). Overall effect of diet (*P* = 0.306; *P* = 0.226), trailering (*P* = 0.011; *P* = 0.172), muscle group (*P* < 0.0001; *P* < 0.0001), diet $\times$ trailering (*P* = 0.258; *P* = 0.596), diet $\times$ muscle group (*P* = 0.014; *P* = 0.945), time $\times$ muscle group (*P* = 0.840; *P* = 0.119) and diet $\times$ time $\times$ muscle group (*P* = 0.071; *P* = 0.450) for panels a and b respectively.

At week 0, pre-trailer MDA of INORG horses was greater in the TB than the GM (*P* = 0.02). Malondialdehyde in the TB of INORG horses then tended to decrease at 1 h post-trailer (*P* = 0.10) and increased at 24 h post-trailer (*P* = 0.03; Fig. 1a). There was no change in GM MDA of INORG horses, which resulted in greater MDA in the TB than GM 24 h after trailering in INORG horses (*P* = 0.0001). In CTM horses, TB MDA concentration was not affected by trailering, but GM MDA increased 24 h after trailering (*P* = 0.001; Fig. 1a). Due to the different changes in muscle groups between CTM and INORG horses, MDA was greater in the GM of CTM compared to INORG horses (*P* = 0.003) and tended to be greater in the TB of INORG compared to CTM horses (*P* = 0.10) 24 h post-trailering. At week 12, MDA concentration was greater in the TB than the GM (*P* < 0.0001). A trend for a time $\times$ muscle group interaction (*P* = 0.06) indicated that MDA concentration increased in the GM at 1 h post-trailer (*P* = 0.002) and decreased from 1 to 24 h post-trailer (*P* = 0.05; Fig. 1b) but MDA was unaffected by trailering in the TB. Diet did not influence MDA responses to trailering at week 12.

**Figure 2:**
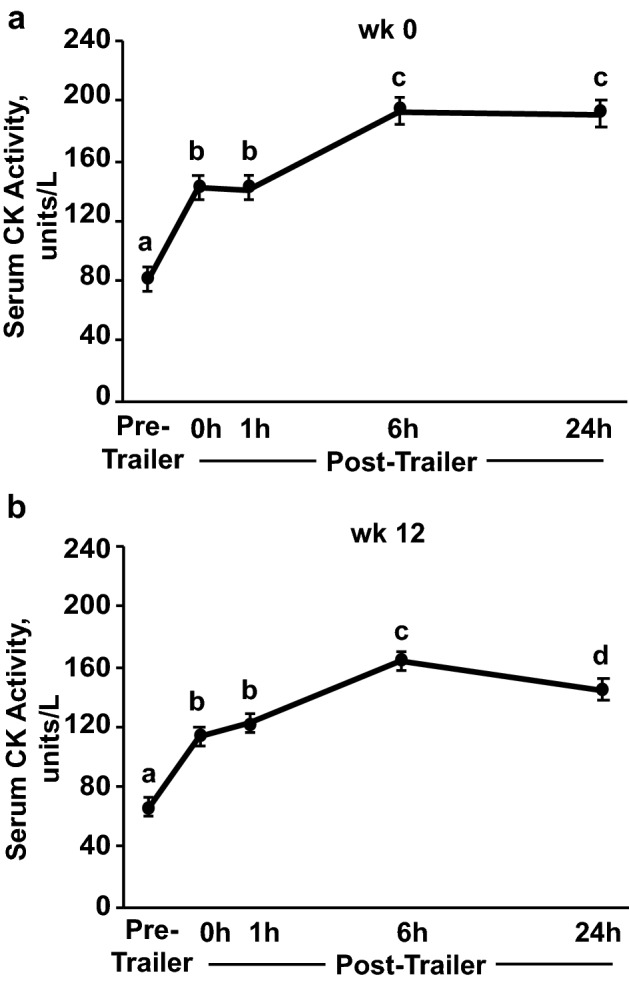
Serum creatine kinase (CK) activity before (Pre-Trailer), and 0, 1, 6, and 24 h after (0 h, 1 h, 6 h, and 24 h, respectively) a 1.5-h trailer stressor. Trailer stressors occurred before (week 0; **a**) and after (week 12; **b**) 12 weeks of submaximal exercise training in yearling horses (n = 16). Due to lack of effect of diet, dietary treatments have been combined. Overall effect of trailering (*P* < 0.0001; *P* < 0.0001) for panels **a** and **b**, respectively. ^a–d^Time points lacking common letters differ (*P* ≤ 0.05).

Following both trailer stressors, serum CK activity increased immediately after trailering (0 h; *P* < 0.0001) and remained elevated through 24 h post-trailer (*P* < 0.0001; Fig. 2) for both treatments. At week 12 but not week 0, CK activity decreased from 6 to 24 h post-trailer (*P* = 0.007; Fig. 2b). Serum CK activity following trailering was unaffected by diet.

#### Antioxidant activities

**Figure 3:**
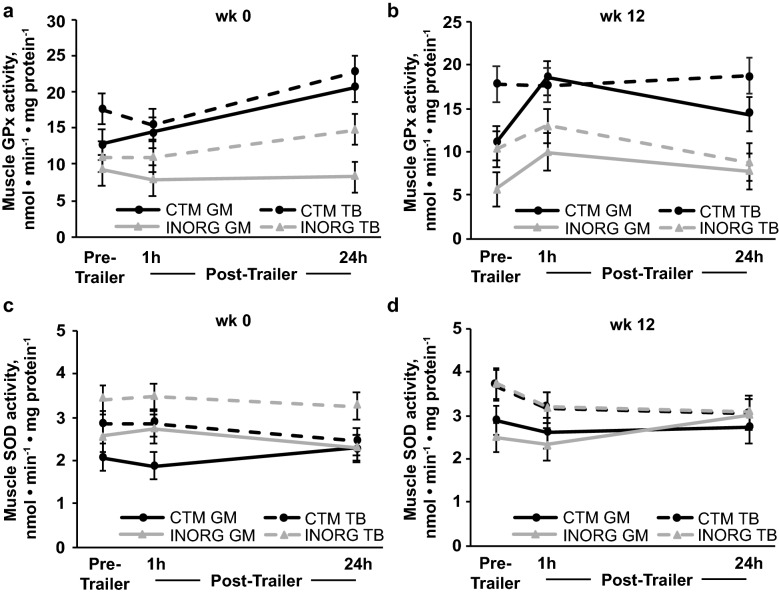
Gluteus medius (GM) and triceps brachii (TB) glutathione peroxidase (GPx; **a**,**b**) and superoxide dismutase (SOD; **c**,**d**) activites before (pre-trailer), and 1 (1 h) and 24 h (24 h) after a 1.5-h trailer stressor. Trailer stressors occurred before (week 0; **a**,**c**) and after (week 12; **b**,**d**) 12 weeks of submaximal exercise training in yearling horses supplemented with either complexed trace minerals (CTM; n = 8) or inorganic trace minerals (INORG; n = 8). Overall effect of diet (*P* = 0.0002; *P* = 0.0003; *P* = 0.061; *P* = 0.941), trailering (*P* = 0.055; *P* = 0.011; *P* = 0.697; *P* = 0.662), muscle group (*P* = 0.005; *P* = 0.001; *P* < 0.0001; *P* = 0.011), diet × trailering (*P* = 0.370; *P* = 0.522; *P* = 0.737; *P* = 0.777), diet × muscle group (*P* = 0.307; *P* = 0.979; *P* = 0.507; *P* = 0.367), time × muscle group (*P* = 0.941; *P* = 0.069; *P* = 0.701; *P* = 0.501) and diet × time × muscle group (*P* = 0.533; *P* = 0.485; *P* = 0.415; *P* = 0.443) for panels (**a**–**d**), respectively.

Overall, GPx activity was greater for CTM than INORG horses in both muscle groups (*P* ≤ 0.0003; Fig. 3a,b), and greater in the TB than the GM (*P* ≤ 0.005) throughout both trailer stressors. In response to trailering at week 0, a trend for an effect of trailering (*P* = 0.06) suggested that GPx activity increased 24 h after trailering (*P* = 0.03; Fig. 3a) in both muscle groups. At week 12, a trend for a trailering $\times$ muscle group interaction (*P* = 0.07) suggested that GPx activity increased in the GM 1 h after trailering (*P* = 0.0004) and remained elevated through 24 h post-trailer (*P* = 0.02), but did not change in response to trailering in the TB (Fig. 3b).

Muscle SOD activity tended to be greater for INORG than CTM horses throughout the week 0 trailer stressor in both muscle groups (*P* = 0.08; Fig. 3c) but was not different between dietary treatments during the week 12 trailer stressor (Fig. 3d). Activity of SOD was greater in the TB than the GM throughout both trailer stressors (*P* ≤ 0.0008) but was not affected by trailering at week 0 or 12.

At week 0, GPx activity in whole blood tended to increase from pre-trailer to 1 h post-trailer (*P* = 0.10) and continued increasing to 6 h post-trailer (*P* = 0.007) but returned to pre-trailer levels by 24 h post-trailer (Supplemental Figure S1). Blood GPx activity at week 12 and blood SOD activity at week 0 and 12 were not affected by dietary treatment, trailering, or their interaction (Supplemental Figure S1).

#### Muscle antioxidant gene expression

**Figure 4:**
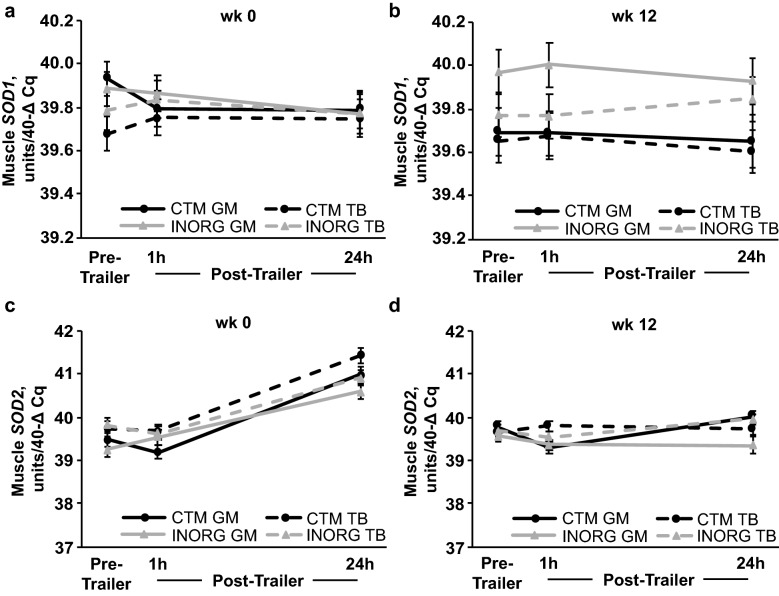
Gluteus medius (GM) and triceps brachii (TB) *Cu–Zn superoxide dismutase* (*SOD1*; **a**,**b**) and *Mn superoxide dismutase* (*SOD2*; **c**,**d**) expression before (pre-trailer), and 1 (1 h) and 24 h (24 h) after a 1.5-h trailer stressor. Trailer stressors occurred before (week 0; **a**,**c**) and after (week 12; **b**,**d**) 12 weeks of submaximal exercise training in yearling horses supplemented with either complexed trace minerals (CTM; n = 8) or inorganic trace minerals (INORG; n = 8). Overall effect of diet (*P* = 0.468; *P* = 0.031; *P* = 0.907; *P* = 0.221), trailering (*P* = 0.607; *P* = 0.922; *P* < 0.0001; *P* = 0.016), muscle group (*P* = 0.138; *P* = 0.050; *P* = 0.003; *P* = 0.029), diet × trailering (*P* = 0.932; *P* = 0.864; *P* = 0.569; *P* = 0.717), diet × muscle group (*P* = 0.403; *P* = 0.189; *P* = 0.784; *P* = 0.052), trailering × muscle group (*P* = 0.505; *P* = 0.881; *P* = 0.371; *P* = 0.113) and diet × trailering × muscle group (*P* = 0.598; *P* = 0.782; *P* = 0.233; *P* = 0.006) for panels (**a**–**d)**, respectively.

At week 0, *SOD1* expression was not affected by diet, trailering, or any interactions, and was not different between muscle groups (Fig. 4a). At week 12, *SOD1* expression was not affected by trailering but was greater for INORG than CTM horses (*P* = 0.03) and was greater in the GM than the TB (*P* = 0.05; Fig. 4b) throughout the trailering stressor.

Expression of *SOD2* increased in response to the week 0 trailer stressor at 24 h post-trailer in both muscle groups of all horses (*P* < 0.0001; Fig. 4c). A trend for a diet $\times$ time interaction suggested that *SOD2* expression was greater for CTM than INORG horses at 24 h post-trailer (*P* = 0.008). During the week 12 trailer stressor, *SOD2* expression decreased at 1 h post-trailer in the GM for CTM horses (*P* = 0.004; Fig. 4d), and then increased from 1 to 24 h post-trailer (*P* < 0.0001) resulting in greater *SOD2* expression for CTM than INORG horses in the GM at 24 h post-trailer (*P* = 0.0007). In the TB, INORG *SOD2* expression increased 24 h after trailering (*P* = 0.02), but was not different from pre-trailer or CTM horses at 24 h post-trailer.

## Discussion

The present study examined the effects of trace mineral supplementation and trailering on oxidative stress and antioxidant status in growing horses undergoing a submaximal exercise training program. Compared to supplementation with inorganic trace minerals, complexed trace minerals conferred few advantages to unstressed, exercising yearling horses regarding oxidative stress and muscle perturbation. However, CTM horses showed improved muscle GPx activity both prior to and during a 1.5-h trailer stressor, as well as an increase in resting muscle SOD activity through 12 weeks of exercise training. Regardless of diet, 12 weeks of exercise training and growth resulted in a decrease in resting markers of oxidative stress (MDA) and muscle permeability (serum CK) but no significant change in antioxidant status. Additionally, the 1.5-h trailer stressor utilized in this study resulted in an increase in serum CK activity, indicating its usefulness as a muscle perturbation model in young horses.

Muscle MDA concentration was used as a marker of oxidative stress in the current study. Resting MDA concentrations (pre-trailer) were higher for INORG than CTM horses at week 0, but decreased for INORG horses by week 12. The elevated muscle MDA concentration for INORG horses at week 0 was not mirrored by an elevated resting serum CK activity, suggesting that the difference in MDA concentration was not physiologically significant enough to result in muscle perturbation at rest. Alternatively, a different marker of muscle damage may be useful in examining resting muscle health, as serum CK activity is typically used as a marker of muscle damage following an acute stressor.

Creatine kinase is an enzyme found primarily in muscle, and its activity in the serum has been shown to increase in response to strenuous exercise as a result of altered muscle cell membrane permeability^8^ or muscle damage^9^. While serum CK activity of horses in the present study remained within normal reference ranges, horses exhibited an increase in serum CK activity in response to each trailer stressor. This was expected based on previous literature^10,11^ and suggested that 1.5 h of trailering caused sufficient perturbation to muscle cells in this study. While not statistically compared, it is important to note that serum CK peaked around 200 units/L following the week 0 trailer stressor, but only increased to around 160 units/L following the week 12 trailer stressor. Combined with the decrease in serum CK activity at 24 h post-trailering at week 12, and the lack of effect of trailering on muscle MDA, the data indicate that 12 weeks of exercise training and growth in yearlings mitigated transportation-induced oxidative stress and muscle perturbation. Unfortunately, one limitation of this study is the lack of a non-exercised control group. Therefore, it is not possible to distinguish the effects of exercise from those of growth. More research is needed to determine the individual impacts of growth and training on skeletal muscle redox response to trailer stress in young horses. Additionally, the isolated effects of repeated muscle micro-tissue collections on serum CK in horses should be defined.

Muscle SOD activity was similar amongst all horses through 12 weeks of dietary treatments, prior to enrollment in an exercise training program (prior study; Supplemental Table S2). However, following 12 weeks of exercise and growth (current study), muscle SOD activity increased in both muscle groups of CTM horses, but not INORG horses. Improvements to the energy-producing cells of skeletal muscle, mitochondria, following submaximal exercise training has been demonstrated in 2-year-old Quarter Horses by an increase in intrinsic mitochondrial capacities of complex I and complex II of the electron transport system following 9 weeks of submaximal exercise training^12^. While the efficiency of mitochondrial coupling also increased^12^, which would decrease the loss of electrons during ATP production, mitochondria remain a source of potentially damaging by-products of energy production, reactive oxygen species, during exercise^13^. Therefore, antioxidant status becomes particularly important during an exercise training program. As such, a lack of increase in SOD activity exhibited by INORG horses coupled with consistently lower GPx activity may have negative implications for young performance horses. This should be further investigated utilizing a more strenuous exercise regime or acute stressor.

In the current study, SOD activity throughout the week 0 trailer stressor tended to be higher for INORG than CTM horses, whereas GPx activity was higher for CTM than INORG horses in both muscle groups at all sampling times. These results are in contrast to previously published literature in food animals showing an increase in SOD activity in various organs following complexed trace mineral supplementation^4,5^. A similar inverse relationship between SOD and GPx expression and activity has been shown in horses supplemented with dietary antioxidants^14,15^, which has been suggested to indicate a preference for use of the GPx over the SOD antioxidant system in horses with more favorable antioxidant or selenium (Se) status. The link between CTM supplementation and increased GPx activity in the present study is not clear, as GPx is a selenoenzyme, and Se was not one of the complexed trace minerals supplemented. However, research has shown an increase in GPx activity in the serum and liver of rats following Zn supplementation^16^. The increased GPx activity purportedly resulted from increased Se availability in tissues in response to Zn supplementation. Additionally, Cu deficiency has been shown to reduce GPx activity in rats despite maintenance of normal dietary and tissue Se levels, suggesting a Se-independent influence of Cu on GPx activity^17^. Therefore, bioavailability of dietary trace minerals in the CTM horses may have influenced GPx activity in a Se-dependent or independent manner. More research is needed to determine the effects of individual complexed trace minerals on GPx activity.

At each week, muscle GPx activity increased following the trailer stressor. At week 12, muscle GPx was elevated sooner and returned to pre-trailer values by 24 h after trailering, further supporting the idea that 12 weeks of exercise training improved the response to a 1.5 h trailer stressor in yearlings. It is interesting to note that although CTM horses had significantly higher resting GPx activity compared to INORG horses, CTM horses still exhibited an increase in activity in response to trailering. This may result in part from the fact that GPx can be activated by signals other than increased cellular oxidant concentration. For example, GPx is activated by nuclear factor (NF) κβ^18^. Activation of NFκβ after acute exercise has often been attributed to increased ROS production during exercise, but may also be influenced by pro-inflammatory cytokines and other intermediates independently of exercise-induced ROS^19^. Therefore, it is possible that increases in stress signals independent of ROS caused activation of NFκβ and therefore an increase in GPx activity in response to trailering.

Following the week 0 trailer stressor, whole blood GPx activity increased 1 h post-trailering. These results are contrary to studies that have shown either no change^20^ or a decrease in systemic GPx activity in horses following transportation^21^, but similar to results showing an increase in muscular and systemic GPx activities following an acute exercise bout^15^. Differences between our results and previous studies of transportation stress may have arisen due to differences in length of transportation or the age of horses in the studies. There was no change in blood GPx activity following the week 12 trailer stressor, further suggesting that 12 weeks of exercise training attenuated redox response to the trailer stressor.

Activity of SOD tended to be lower for CTM than INORG horses throughout the trailer stressor at week 0, and was accompanied by a greater *SOD2* expression response to trailering for CTM horses. At week 12, growing horses supplemented with dietary complexed trace minerals showed similar SOD activity and expression to INORG horses throughout the trailer stressor. Therefore, 12 weeks of exercise training in growing horses in addition to CTM supplementation ultimately led to similar SOD activities between treatments, but higher sustained GPx activity for CTM horses.

At both sampling intervals, the trailer stressor induced an increase in *SOD2* expression at 24 h post-trailering. Since ROS and inflammatory cytokines are known to regulate transcription of *SOD2*^22,23^, the increase in *SOD2* expression at 24 h post-trailer again suggests that redox balance of the horses may have been affected by the trailer stressor. As such, we would have expected an increase in oxidative stress as indicated by an increase in MDA concentration at week 12. An alternative marker to MDA, such as protein carbonyls or lipid hydroperoxides, or different sampling intervals may have revealed a more prevalent oxidative stress response. Conversely, an increase in inflammatory cytokine signaling following trailering could have also been responsible for the observed increase in *SOD2* expression.

The GM is composed of a greater percentage of fast-twitch, non-oxidative fibers when compared to the TB^24^. Additionally, the TB has been found to have higher indices of mitochondrial density^12^. In the present study, resting MDA concentration, GPx activity, SOD activity, and *SOD2* expression were higher in the TB than the GM. These data suggest that in addition to having a lower percentage of fast-twitch, non-oxidative fibers and higher mitochondrial density, the TB also has a higher antioxidant capacity when compared to the GM. Interestingly, *SOD1* expression was higher in the GM than the TB. Similarly, the semimembranosus, which contains a low proportion of type I and type IIa muscle fibers, had the highest expression of *SOD1* in a study of beef cattle^25^. These data in conjunction with our results suggest that *SOD1* expression is higher in less oxidative muscle groups. Therefore, the propulsive and stabilization functions of the GM and TB, respectively, are reflected by differences in fiber type and mitochondrial density, as well as by differences in oxidative by-products and antioxidant activity and expression.

## Conclusion

In the present study of yearling horses, trailering caused an increase in CK activity and muscle MDA concentrations, and changes in antioxidant defense systems indicative of alterations in redox homeostasis. Growing horses showed decreased systemic markers of muscle damage and resting MDA concentrations, increased resting SOD activity and *SOD2* expression in the GM, and attenuated responses of MDA, CK and blood GPx activity associated with trailer stress through 12 weeks of submaximal exercise training. Supplementation with complexed trace minerals increased muscle GPx activity at rest and in response to trailer stress, ultimately leading to preferential utilization of the GPx system over the SOD system in response to oxidative stress in young untrained horses. However, after 12 weeks of exercise training, horses supplemented with CTM exhibited increased muscle SOD activity, and maintained significantly higher muscle GPx activity. Therefore, complexed trace minerals may be a useful tool for mitigating oxidative stress to maintain muscle health in young equine athletes.

## Methods

### Horses

This study was reviewed and approved by the Texas A&M Institutional Animal Care and Use Committee (IACUC; 2016–0294). All experiments were performed in accordance with IACUC guidelines and regulations. Sixteen yearling Quarter Horses (7 fillies and 9 colts) with a mean age of 9.7 mo (SD 0.7) and BW of 295 kg (SEM 5) were used in this study. Horses were housed in paddocks by sex (0.53 and 0.72 ha for colts and fillies, respectively) at the Texas A&M University Freeman Arena Facility in College Station, TX.

### Dietary treatments

Horses were grouped by age, sex, and BW and randomly assigned to receive custom concentrates containing either complexed (**CTM**; Zn-Met, Mn-Met, Cu-Lys, and Co-glucoheptonate; n = 8) or inorganic (**INORG**; CuSO_4_, ZnSO_4_, MnSO_4_ and CoCO_3_; n = 8) supplemental trace minerals. Horses were enrolled in a separate but related study prior to the beginning of this study^26^. Therefore, all horses had received the experimental treatments for 12 weeks prior to the current experiment. Horses were allocated to separate pens by sex and had ad libitum access to coastal Bermudagrass hay and water. Hay intake per horse per day was estimated by the following formula:

$$\frac{{Total\;hay\;offered\;in\;the\;pen\;per\;day \left( {{\text{kg}}} \right) - total\;hay\;refused\;in\;the \;pen\;per\;day \left( {{\text{kg}}} \right)}}{Number\;of\;horses\;in\;the\;pen}.$$

Concentrate was offered at 1.25% BW/day (DM basis). Horses received grain meals individually in stalls (3.7 $\times$ 3.7 m) split equally into two meals fed at 0700 and 1700. Refusals were monitored and recorded daily to calculate intake of concentrate. Total daily intake of estimated hay intake plus concentrate intake was 2.0% BW/day (DM basis). Diets were formulated to maintain a BCS of 5 to 6 according to the Henneke body condition scoring system^27^, and to meet all requirements for growing horses undergoing light exercise^28^. Throughout the study, BW of horses was recorded weekly using a livestock scale accurate to 1 kg and heart girth, body length, hip height, and wither height measurements were collected at week 0 and 12 to assess growth. Heart girth and body length were measured using a flexible measuring tape. Hip height and wither height were measured using an altitude stick. Measurements were collected by the same two individuals throughout the study, and averaged at each time point to obtain heart girth, body length, hip height, and wither height values.

All feeds were analyzed prior to beginning the study. Concentrates were analyzed by Equi-Analytical Laboratories (Ithaca, NY), and hay was analyzed by Elk River Forage Lab (Elk River, MN) using standard analytical methods (Table 4). Actual intake of each mineral of interest is presented in Supplemental Table S3.

**Table 4:** Nutrient composition of bermudagrass hay, inorganic (INORG) concentrate and complexed trace mineral (CTM) concentrate.

Nutrient^a^	Bermudagrass Hay	INORG	CTM
Digestible energy (Mcal/kg)	1.97	2.72	2.78
Crude fat (%)	2.3	7.7	7.6
Crude protein (%)	12.5	19.3	20.0
Neutral detergent fiber (%)	66.0	40.0	36.9
Acid detergent fiber (%)	35.5	25.9	24.5
Calcium (%)	0.38	0.94	1.20
Phosphorus (%)	0.26	0.92	1.05
Selenium (mg/kg)	0.39	0.73	0.97
Zinc (mg/kg)	29	142	210
Manganese (mg/kg)	187	157	212
Copper (mg/kg)	8	50	58
Cobalt (mg/kg)	0.00	6.14	8.04

^a^Values presented on a 100% DM basis.

### Exercise

Horses received no forced exercise prior to the beginning of this study. Beginning at week 0, horses were enrolled in a 12-week submaximal exercise program. To facilitate trailer transportation and sample collection, horses were further divided into eight collection groups each with equal representation of dietary treatments. Exercise training, sampling and trailering were initiated in one pair of horses per day over two weeks with four consecutive days of exercise training, trailering and sample collection initiation each week. Exercise was designed to achieve light work as defined by the NRC^28^, and consisted of 12 min of walking, 15 min of trotting, and 3 min of cantering in a free-stall exerciser (30 min total/day) 5 days/week. Each gait was performed daily for an equal amount of time in both directions, and horses alternated starting the exercise bout clockwise or counterclockwise. The speed of each gait was progressively increased throughout the study as horses grew to ensure that all horses remained in the intended gait. Gait speeds started at 1.1 m/s for the walk, 2.5 m/s at the trot and 5.0 m/s at the canter at the beginning of training and were increased to 1.2 m/s at the walk, 3.0 m/s at the trot and 5.4 m/s at the canter by the end of the 12-week exercise training program.

### Trailer stressor

Before beginning exercise training (week 0), and after 12 weeks of exercise training (week 12), horses were trailered for 1.5 h to assess the effects of trailer stress on antioxidant activity and markers of oxidative stress and muscle damage. Horses were trailered in pairs with equal representation of dietary treatements, tied in the front-most stall of a gooseneck stock trailer. Trailering was performed between 0800 and 1000, on the same route, and by the same driver for all trailering sessions. The average temperatures during trailer sessions were 14.5 ± 2.2 °C and 21.2 ± 2.3 °C at week 0 and 12, respectively. The average humidity during trailer sessions were 59.7 ± 7.0% and 78.1 ± 4.3% at week 0 and 12, respectively.The trailering route was approximately 113 km in distance, and consisted of approximately 81 km of highway with no traffic stops, and 32 km of driving in the city with light traffic and intermittent stops at traffic lights.

### Sample collection

Muscle and blood samples were collected surrounding each 1.5-h trailer stressor at week 0 and 12. Muscle samples were collected before, and at 1 and 24 h post-trailering for analysis of muscle MDA concentration, GPx and SOD activities, and *SOD* gene expression. Muscle tissue samples were collected from the GM and TB using a tissue collection procedure as previously described^15^. Briefly, horses were sedated with detomidine hydrochloride prior to beginning tissue collection procedures. The collection areas were clipped, scrubbed with a 7.5% povidone-iodine solution, and then rinsed with a 70% ethanol solution. The tissue collection sites were desensitized with 0.5 mL of 2% lidocaine and a 14-gauge needle was used to create the initial puncture through the skin. Tissue was collected using a 14-gauge, 9-cm tissue collection needle inserted to a depth of 3.5 cm. The tissue collection site altered between left and right muscle groups at each sampling interval. Samples obtained from the same side of the horse were obtained approximately 2 cm from the previous insertion site. At each sampling interval, approximately 75 mg (wet weight) of tissue was placed in 1 mL of RNALater (Invitrogen, Carlsbad, CA) and stored at − 20 °C until gene expression analysis was performed. An additional 300 mg (wet weight) of muscle tissue was flash frozen in liquid nitrogen and stored at − 80 °C until enzymatic activity analyses were performed. Flash frozen muscle was cryopulverized into a fine powder for evaluation for SOD and GPx activities and MDA concentration.

Blood samples were collected prior to sedation before trailering, immediately after, and 1, 6 and 24 h after trailering for analysis of serum CK activity and whole blood GPx and SOD activities. At each sampling interval, approximately 15 mL of blood was collected into evacuated containers, containing either no anticoagulant for harvesting of serum or sodium heparin for harvesting of whole blood. Serum samples remained at 25 °C and plasma samples were placed on ice for approximately 1 h prior to processing. Samples were then centrifuged at 3,000 × *g* for 10 min at 4 °C, and serum and whole blood were harvested and stored at − 80 °C until analysis.

### Malondialdehyde concentration

Muscle samples were evaluated for MDA concentrations per manufacturer instructions using a commercially available kit (Northwest Life Science Specialties, LLC, Vancouver, WA) as previously described^29^. Previously cryopulverized muscle powder was diluted 1 mg tissue (wet weight) to 10 uL assay buffer provided in the kit and sonicated 3 times for 3 s each on ice, and then centrifuged at 11,000 × *g* for 10 min at 4 °C. Homogenate supernatants were collected and stored at − 80 °C until analysis. Samples were analyzed in triplicate with an intra-assay CV of 3.6% and an inter-assay CV of 1.4%. Samples for analysis of MDA concentration were within the standard range of the assay (0.1 to 10 µM). Concentration of MDA was normalized to sample total protein concentration, quantified using a Coomassie Protein Assay kit (Thermo Fisher Scientific).

### Enzyme activities

Serum samples were analyzed for CK activity as a marker of muscle damage^30^ using a commercially available kit, following manufacturer instructions (CK Liqui-UV; EKF Diagnostics, Boerne, TX). Samples were analyzed in triplicate with an intra-assay CV of 3.9% and an inter-assay CV of 9.8%. All samples were within the linear range of the assay (1 to 1200 U/L).

Whole blood and muscle samples were analyzed for SOD and GPx activities as measures of antioxidant status using commercially available kits (Cayman Chemical Company, Ann Arbor, MI). Muscle tissue that had been previously cryopulverized and stored at − 80 °C was diluted 1 mg tissue (wet weight) to 40 uL extraction buffer (1 mM EGTA, 210 mM mannitol, 70 mM sucrose, pH 7.2), and 1 mg tissue (wet weight) to 80 uL extraction buffer for GPx and SOD analysis, respectively. Diluted samples were sonicated using a sonic dismembrator 3 times for 3 s each on ice, and then centrifuged at 10,000 × *g* for 15 min at 4 °C. Homogenate supernatants were collected and stored at − 80 °C until analysis. Activity of GPx was evaluated in whole blood diluted 1:34 (v/v), and SOD activity was evaluated in whole blood diluted 1:150 (v/v) with assay buffer provided in the respective kits to ensure sample concentration was within the standard curve of assays. Whole blood and muscle samples were analyzed per manufacturer instructions in triplicate for GPx activity and in duplicate for SOD activity. Intra-assay CV was 4.5, 3.5, 4.8, and 3.6% and inter-assay CV was 10.3, 12.1, 9.3, and 10.1% for muscle GPx, muscle SOD, blood GPx, and blood SOD activities, respectively. Samples for analysis of GPx and SOD activities were within the dynamic range of the assays (50 to 344 nmol • min^−1^ • mL^−1^ for GPx, 0.005 to 0.05 U/mL for SOD). Muscle homogenate and blood sample total protein was quantified using a Coomassie Protein Assay kit (Thermo Fisher Scientific), and all antioxidant enzyme activities were normalized to sample total protein concentration.

### Gene expression

Muscle was examined for mRNA expression of antioxidant enzymes *Cu–Zn superoxide dismutase* (*SOD1*) and *Mn superoxide dismutase* (*SOD2*). Briefly, approximately 75 mg (wet weight) tissue was homogenized in TRIzol (Invitrogen) using a bead homogenizer 4 times for 30 s intervals. 1-Bromo-3-cholorpropane was used to extract RNA from samples followed by isolation on a spin column according to manufacturer instruction (Purelink RNA Mini-kit; Invitrogen). Samples were treated with RNase-free DNase on the column during RNA extraction to remove any DNA contamination. Spectrophotometry was used to assess RNA concentration and optical density (OD) ratios on a 16-well plate. In all cases, RNA yield was greater than 15 ng/uL and OD_260/280_ was greater than 1.5. Samples were reverse transcribed using the Superscript II Reverse Transcription kit (Invitrogen). Two hundred ng RNA was reverse transcribed to cDNA for the majority of the samples. However, 29 samples had a low (< 25 ng/uL) RNA yield; for those samples, 120 ng RNA was reverse transcribed. The cDNA from reverse transcription was amplified with SYBR Green PCR master mix and the appropriate forward and reverse primers (Supplemental Table S4). Thermal cycling parameters included a denaturation step of 95 °C for 5 min followed by 40 cycles at 15 s at 95 °C and 1 min at 60 °C. Efficiency of PCR for each primer set was determined using a standard curve of pooled cDNA^31^. A Cycle of Quantification (**Cq**) value was determined for each sample via PCR. Data for each gene were normalized using the geomean of *succinate dehydrogenase A* (***SDHA***), *hypoxanthine phosphoribosyltransferase 1* (***HPRT1***), and *beta-2 microglobulin* (***B2M***) as reference genes to account for total cDNA in each sample. The resulting ΔCq was calculated by subtracting Cq_geomean_ from Cq_gene of interest_. In figures, gene expression is represented as 40 − ΔCq, in which 40 is the total number of cycles ran. Fold changes in gene expression are also reported and are calculated using the formula 2^−ΔΔCq^, in which − ΔΔCq is the ΔCq_time of interest_ − ΔCq_PreT_ (before trailer stressor) for each horse^32^.

### Statistical analyses

Investigators remained blinded to dietary treatments through statistical analysis. Differences in serum CK activity, muscle and blood SOD and GPx activities, muscle *SOD1* and *SOD2* expression, and muscle MDA concentration were analyzed using the MIXED procedure of SAS 9.4 (SAS Institute, Inc, Cary, NC) with repeated measures. The responses to diet, exercise and growth over time (week 0 and 12 pre-trailer) were analyzed separately from responses to trailering at each week. Additionally, the response to trailering at week 0 was analyzed separately from the response to trailering at week 12. Data were tested for normality and then log-transformed before analysis if not normally distributed. For pre-trailer responses to diet and exercise, diet (CTM and INORG), time (week 0 and 12), muscle group (GM and TB), and all interactions were included in the model as fixed effects, and horse within diet was a random effect. Sex and collection group were also included in the model and removed if not significant. For responses to trailering, the model included diet, trailering (pre-trailer, 1 and 24 h post-trailer for muscle; pre-trailer, 0, 1, 6, and 24 h post-trailer for blood), muscle group, and all interactions as fixed effects, and horse within diet as a random effect. In cases where either week 0 or pre-trailer values were significantly different between treatments, the covariate was tested. If the covariate was not significant, it was removed from the model. All data are expressed as least squared means ± SEM. Significance was considered at *P* ≤ 0.05, and trends were acknowledged at *P* ≤ 0.10.

## Supplementary Information

Supplementary Information

## Data Availability

The datasets generated during and/or analyzed during the present study are available from the corresponding author on reasonable request.
