# Supplementary material for: Complexed trace mineral supplementation alters antioxidant activities and expression in response to trailer stress in yearling horses in training
Source: Sci Rep. 2021 Apr 1;11:7352. doi: 10.1038/s41598-021-86478-7 (PMC8016935; doi:10.1038/s41598-021-86478-7)
Supplement: Supplementary file 1 — Supplementary Information [file 41598_2021_86478_MOESM1_ESM.pdf]

Complexed trace mineral supplementation alters antioxidant activities and expression in response to trailer stress in yearling horses in training

Christine M. Latham, Emily C. Dickson, Randi N. Owen, Connie K. Larson, and Sarah H. White-Springer

Supplemental Table S1 Body weight, body condition score, heart girth, body length, wither height, and hip height in yearlings (n = 16) before (wk 0) and after 12 wk of growth and exercise training. Due to lack of effect of diet, dietary treatments have been combined.

	Week			*P*-Value
Variable	0	12	SEM	Time
Body Weight, kg	293.6	330.6*	6.8	<0.0001
Body Condition Score	6.1	5.5*	0.1	<0.0001
Heart girth, cm	148.8	156.4*	1.3	<0.0001
Body Length, cm	144.8	149.4*	1.2	<0.0001
Wither Height, cm	51.8	53.4*	0.2	<0.0001
Hip Height, cm	54.3	56.0*	0.3	<0.0001

*Within a row, mean differs from wk 0 ($P \leq 0.05$).

Supplemental Table S2. Glutathione peroxidase (GPx) and superoxide dismutase (SOD) activities in the gluteus medius (GM) and triceps brachii (TB) and serum creatine kinase (CK) activity at -12 and -6 wk relative to the current study. Horses were supplemented with either complexed trace minerals (CTM; n = 8) or inorganic trace minerals (INORG; n = 8). Week -12 is pre-supplementation and wk-6 is after 6 wk of dietary treatment but 6 wk before horses entered the exercise training program described in the current study.

						P-Value		
Enzyme	Muscle	Diet	wk-12	wk-6	SEM	Diet	Time	Diet × Time
Muscle GPx activity, nmol • min^{-1} • mg $protein^{-1}$	GM	CTM	12.06	12.68	3.90	0.263	0.319	0.407
		INORG	13.90	20.65				
	TB	CTM	22.23	16.28	4.68	0.960	0.309	0.749
		INORG	21.08	17.94				
Muscle SOD activity, nmol • min^{-1} • mg $protein^{-1}$	GM	CTM	2.74	3.90	0.45	0.134	0.206	0.248
		INORG	3.99	4.05				
	TB	CTM	3.38	3.84	0.47	0.366	0.147	0.692
		INORG	3.71	4.51				
Serum CK activity, units/L	-	CTM	63.59	66.57[a]	5.75	0.093	0.032	0.084
	-	INORG	66.46	90.86[b]*				

*Within a row, mean differs from wk-12 ($P \leq 0.05$).
[a,b]Within a column, differing letters indicate CTM differs from INORG within the specified variable ($P \leq 0.05$).

Supplemental Table S3. Daily trace mineral intake from concentrate for horses supplemented either complexed trace minerals (CTM; n = 8) or inorganic trace minerals (INORG; n = 8)

Trace mineral[1]	CTM[2]	INORG[3]
**Zn, mg/kg BW**	2.6	1.8
**Mn, mg/kg BW**	2.6	2.0
**Cu, mg/kg BW**	0.7	0.6
**Co, mg/kg BW**	0.10	0.08

[1] Values presented on a 100% DM basis.
[2] Zn-Met, Mn-Met, Cu-Lys, and Co-glucoheptonate
[3] $CuSO_4$, $ZnSO_4$, $MnSO_4$ and $CoCO_3$

Supplemental Table S4. Sequence of equine-specific PCR primers used for measurement of *succinate dehydrogenase A (SHDA)*, *hypoxanthine phosphoribosyltransferase 1* (*HPRT1)*, *beta-2 microglobulin* (*B2M)*, *Cu-Zn superoxide dismutase (SOD1)* and *Mn superoxide dismutase* (*SOD2)* mRNA content.

Gene	Primers 5’-3’ (Forward, Reverse)	Slope[1]	Accession Number
SDHA	AAGTCGATGCAAAGTCATGCT	-3.129	DQ402987
	ACCATTCCTCTGTCAAACGTCT		
HPRT1	TCATGGAGTAATTATGGACAGGACT	-3.245	XM_023634464
	TCATAATCCAGCAGGTCAGCAA		
B2M	TTCTATCTTCTGGTCCATACTGACTT	-3.142	NM_001082502
	GAGGTCTCGATCCCACTTAACTAT		
SOD1	AGATAATACACAAGGCTGTACCACT	-3.158	NM_001081826
	ATTGCCCAGGTCTCCAACAT		
SOD2	AGCCTGCACTCAAGTTCAAT	-3.168	NM_001082517
	TCGAAGGAACCAAAGTCACGTT		

[1]Slope of standard curve of primer sequence used for RT-PCR analysis.

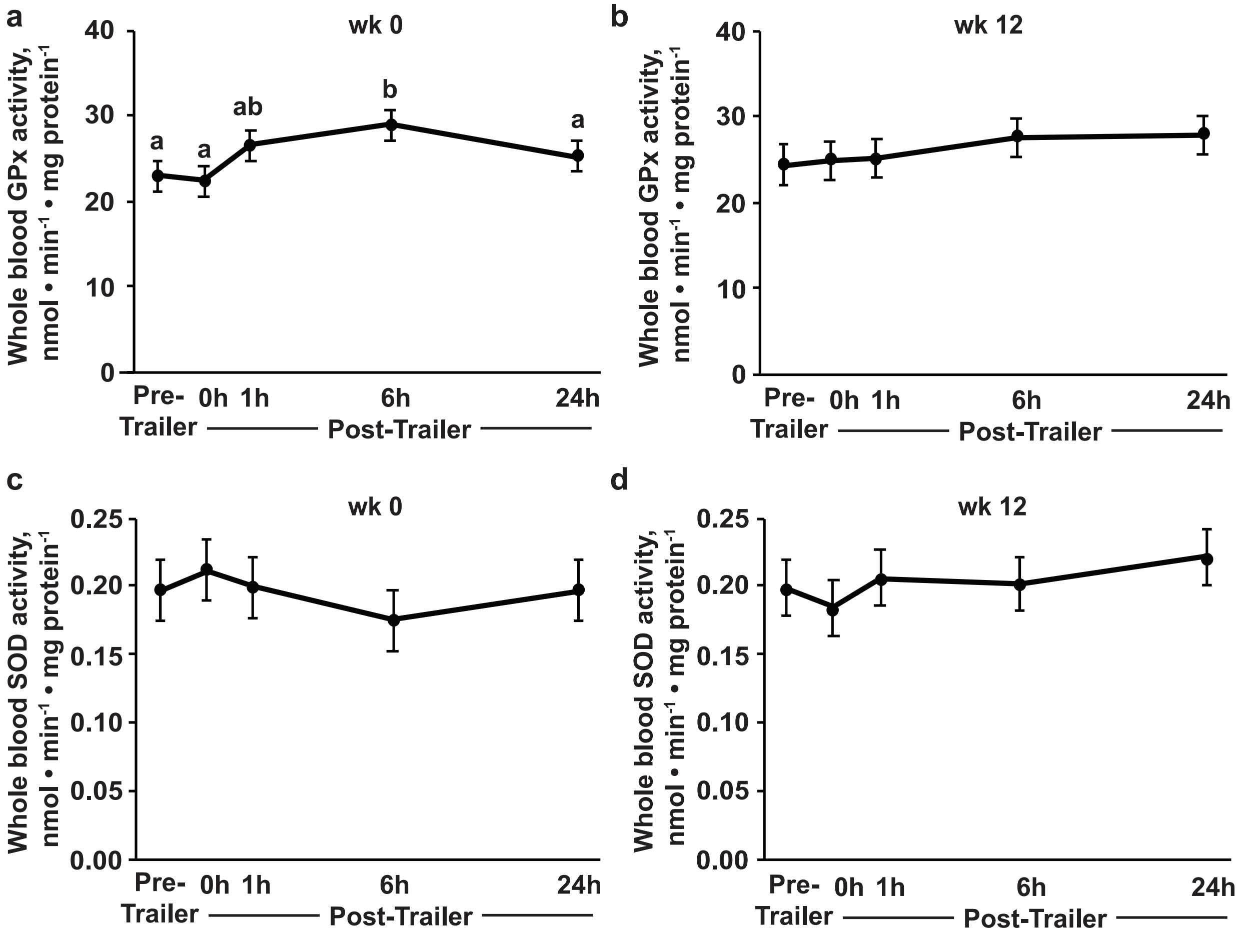

Supplemental Figure S1. Whole blood glutathione peroxidase (GPx; a&b) and superoxide dismutase (c&d) before (Pre-Trailer), and 0, 1, 6, and 24 h after (0h, 1h, 6h, and 24h, respectively) a 1.5-h trailer stressor. Trailer stressors occurred before (wk 0; a&c) and after (wk 12; b&d) 12 wk of submaximal exercise training in yearling horses (n = 16). Due to lack of effect of diet, dietary treatments have been combined. Overall effect of trailering (P = 0.016; P = 0.524; P = 0.238; P = 0.609) for panels a, b, c and d, respectively. [a-d] Time points lacking common letters differ ($P \leq 0.05$).
